# Engaging Users in the Behavior Change Process With Digitalized Motivational Interviewing and Gamification: Development and Feasibility Testing of the Precious App

**DOI:** 10.2196/12884

**Published:** 2020-01-30

**Authors:** Johanna Nurmi, Keegan Knittle, Todor Ginchev, Fida Khattak, Christopher Helf, Patrick Zwickl, Carmina Castellano-Tejedor, Pilar Lusilla-Palacios, Jose Costa-Requena, Niklas Ravaja, Ari Haukkala

**Affiliations:** 1 Discipline of Social Psychology Faculty of Social Sciences University of Helsinki Helsinki Finland; 2 Behavioural Science Group, Primary Care Unit University of Cambridge Cambridge United Kingdom; 3 Communications and Networking Department, School of Electrical Engineering Aalto University Espoo Finland; 4 Department of Entertainment Computing University of Vienna Vienna Austria; 5 Center For Digital Safety And Security Austrian Institute of Technology Vienna Austria; 6 Department of Psychiatry, University Hospital Vall d’Hebron Vall d’Hebron Institute of Research Barcelona Spain; 7 Department of Basic Psychology, Grup de Recerca en Estrès i Salut, Autonomous University of Barcelona Bellaterra Spain; 8 Servicio de Psiquiatría, Hospital Universitari Vall d’Hebron, Departament de Psiquiatria i Medicina Legal Universitat Autònoma de Barcelona Barcelona Spain; 9 Centro de Investigación Biomédica en Red de Salud Mental, Instituto de Salud Carlos III Vall d’Hebron Institut de Recerca Barcelona Spain; 10 Department of Psychology and Logopedics Faculty of Medicine University of Helsinki Helsinki Finland; 11 Helsinki Collegium for Advanced Studies University of Helsinki Helsinki Finland

**Keywords:** health app, mHealth, human-computer interaction, prevention, service design, usability design, intrinsic motivation, reflective processes, spontaneous processes, engagement, self-determination theory, autonomous motivation, gamification, physical activity

## Abstract

**Background:**

Most adults do not engage in sufficient physical activity to maintain good health. Smartphone apps are increasingly used to support physical activity but typically focus on tracking behaviors with no support for the complex process of behavior change. Tracking features do not engage all users, and apps could better reach their targets by engaging users in reflecting their reasons, capabilities, and opportunities to change. Motivational interviewing supports this active engagement in self-reflection and self-regulation by fostering psychological needs proposed by the self-determination theory (ie, autonomy, competence, and relatedness). However, it is unknown whether digitalized motivational interviewing in a smartphone app engages users in this process.

**Objective:**

This study aimed to describe the theory- and evidence-based development of the Precious app and to examine how digitalized motivational interviewing using a smartphone app engages users in the behavior change process. Specifically, we aimed to determine if use of the Precious app elicits change talk in participants and how they perceive autonomy support in the app.

**Methods:**

A multidisciplinary team built the Precious app to support engagement in the behavior change process. The Precious app targets reflective processes with motivational interviewing and spontaneous processes with gamified tools, and builds on the principles of self-determination theory and control theory by using 7 relational techniques and 12 behavior change techniques. The feasibility of the app was tested among 12 adults, who were asked to interact with the prototype and think aloud. Semistructured interviews allowed participants to extend their statements. Participants’ interactions with the app were video recorded, transcribed, and analyzed with deductive thematic analysis to identify the theoretical themes related to autonomy support and change talk.

**Results:**

Participants valued the autonomy supportive features in the Precious app (eg, freedom to pursue personally relevant goals and receive tailored feedback). We identified the following five themes based on the theory-based theme autonomy support: valuing the chance to choose, concern about lack of autonomy, expecting controlling features, autonomous goals, and autonomy supportive feedback. The motivational interviewing features actively engaged participants in reflecting their outcome goals and reasons for activity, producing several types of change talk and very little sustain talk. The types of change talk identified were desire, need, reasons, ability, commitment, and taking steps toward change.

**Conclusions:**

The Precious app takes a unique approach to engage users in the behavior change process by targeting both reflective and spontaneous processes. It allows motivational interviewing in a mobile form, supports psychological needs with relational techniques, and targets intrinsic motivation with gamified elements. The motivational interviewing approach shows promise, but the impact of its interactive features and tailored feedback needs to be studied over time. The Precious app is undergoing testing in a series of n-of-1 randomized controlled trials.

## Introduction

### Background

Lifestyle-related diseases, such as cardiovascular diseases, type 2 diabetes, and cancers, lead to a decrease in quality of life and are the leading causes for years of life lost worldwide [1]. Stretched health care resources struggle with the complications that could, in many cases, be avoided with a physically active lifestyle [2,3]. There is a need for interventions that can effectively support people to achieve the amount of physical activity that is necessary for their health and well-being.

As a consequence, hundreds of thousands of smartphone health apps have emerged for tracking physical activity. With their interactive features, onboard sensors, and associated wearables, smartphones are a natural tool for tracking and self-monitoring activity [4,5]. Individuals tend to carry phones with them and keep them switched on continuously [6], use apps repeatedly for brief moments between other activities [6], and value this possibility for ubiquitous support [7].

Tracking physical activity is indeed a key element of many successful physical activity interventions. Self-regulatory behavior change techniques (BCTs) [8] related to control theory [9], such as self-monitoring, goal setting, action planning, feedback on behavior, and problem solving, have been consistently linked with positive changes in physical activity [10]. The more actively individuals enact these techniques, the more effective interventions are [11-14].

Smartphone apps have shown promise in reducing sedentary behaviors [15,16], but the evidence for increasing physical activity is modest [16-20]. One factor that explains this may be the importance of face-to-face contact for physical activity motivation [21]. For smartphone apps to achieve the same effectiveness as interventions delivered in person, apps should be engaging enough for users to keep returning to receive the necessary support. However, user commitment to smartphone apps is low: 22% of downloaded apps are opened only once [22], and users may spend less time with smartphone apps than computer-delivered interventions [6,23]. To engage users and build sustained motivation and commitment in the process of behavior change, apps need to offer more support than just tracking behaviors.

### Engaging Users in the Behavior Change Process With an App

Engagement in digital interventions is conceptualized in different ways in different research traditions. Within behavioral literature, engagement often refers to the frequency or duration of time spent using a digital service [24-26]. This *summative* engagement, the quantitative metrics of usage time and frequency, is not enough for understanding neither the user experience in the moment-to-moment interaction with the app nor *how* apps support active involvement in the behavior change process and the intervention goals [27-29]. Some studies have found low frequency of usage being as effective as higher frequency [30], and increased usage time can even be a symptom of low usability instead of engaging content [30].

Within the usability and gaming literature, engagement is typically conceptualized as the subjective user experience with the service, including affect, interest, attention, and flow [26]. Studying user experience can provide information on how immersive an app is but does not typically explain cognitive engagement in the process of behavior change—a health app may be entertaining and have good usability but does not necessarily engage the user to reflect their behavior or make them take steps to approach their goals.

In this study, we have defined engagement as active involvement with the behavior change process that the app is aiming for. This includes all the steps the user takes toward behavior change, for instance, by reflecting reasons for change or engaging in planning and monitoring of the behavior in question. This concept of engagement with the behavior change process is close to *effective engagement*, which refers to the level of active involvement that is necessary for the intervention to achieve intended outcomes [27,31].

It is recognized that the effectiveness of a digital service depends on the target behaviors (one-time action or lifestyle change), the BCTs used, and individuals using the service and their subjective user experience [26,31]. We have proposed that the effectiveness of a digital service also depends on the extent to which the individuals get cognitively involved in the process of behavior change: whether interaction with an app encourages users to think about their reasons, capabilities, and opportunities to change and whether the interaction increases their motivation and self-efficacy to change. To examine this concept of active cognitive engagement in the behavior change process, we used the approach widely used in face-to-face behavior change counseling (ie, motivational interviewing).

### Motivational Interviewing

Motivational interviewing is a person-centered counseling method that provides practical techniques for improving intervention engagement and commitment to the behavior change process [32]. In addition to *content-focused* BCTs, practical tools for self-regulation, motivational interviewing offers tools for the interaction quality between the counselor and the client, suggesting several *relational* techniques and methods for fostering the alliance and engaging the client in the process [33,34]. Core elements of motivational interviewing include resolving ambivalence toward behavior change, eliciting reflection and *change talk*, individuals’ self-expressed language in favor of change, and supporting client autonomy [32]. Motivational interviewing uses collaborative and nonauthoritarian interaction to work toward clients’ goals [32].

Face-to-face motivational interviewing and telephone-delivered motivational interviewing have increased exercising and strength training and reduced sedentary time [35,36]. A systematic review of technology-delivered adaptations of motivational interviewing showed promise for a variety of health-related behaviors [37]. However, only two of the computer-based interventions targeted physical activity, and none of the interventions tested smartphone delivery [37].

### Motivational Interviewing Increases Intervention Engagement

Motivational interviewing can lead to improved health outcomes through enhancing participant adherence and intervention engagement. It has improved engagement with various behavior change intervention components, including attendance to behavioral weight loss programs [38], and self-monitoring of food intake and blood glucose [35]. For instance, improved program attendance has led to more comprehensive self-monitoring diaries, which again have enhanced weight loss outcomes [39].

Despite the evidence for improving intervention engagement in face-to-face interventions, motivational interviewing–type motivational support is rarely present in health apps for smartphones. For instance, Pagoto et al [40] screened the hundred most popular apps for weight loss and found that none of them provided BCTs for low adherence and motivation.

One reason for the limited use of motivational interviewing may be its lack of having a coherent theoretical framework, which creates challenges for testing the specific mechanisms of action and linking studies to theoretical discussions in the field [41]. Thus, the self-determination theory has been suggested as the theoretical basis for the method, as it shares core principles with motivational interviewing [41].

### Self-Determination Theory

Motivation research leaning on the self-determination theory [42] has established that the quality of motivation predicts individuals’ physical activity levels [43,44]. Individuals with high autonomous motivation are more likely to engage in regular physical activity than those with externally controlled motivations, such as guilt and shame for not being active or pressure from others. Autonomous motivation consists of the following two elements: (1) motivational regulation that is based on the pleasure of the activity itself (ie, intrinsic motivation) and (2) motivational regulation that is guided by goals that are separate from the behavior but in line with the person’s values and identity (ie, identified or integrated regulation). Promoting these different forms of autonomous motivation may require different intervention strategies. Individuals are also more likely to engage in active self-regulation when their motivation for physical activity is autonomous [45-47]. Self-determination theory–based interventions address autonomous motivation by aiming to satisfy the psychological needs of autonomy, competence, and relatedness [48].

*Autonomy* refers to the freedom to organize one’s own experiences and behavior in accordance with one’s integrated sense of self [42]. It can be supported by, for instance, offering choice and a meaningful rationale of the relevance of the behavior, by respecting and acknowledging individuals’ viewpoints, and by avoiding controlling or guilt-inducing acts and language.

Experiences of *competence* can be offered through clear instructions and expectations, collaborative goal setting, and optimally challenging tasks, providing tailored strategies and feedback as well as guidance and skills training. Competence is closely related to Bandura’s concept of self-efficacy [49], which describes an individual’s perceptions of their own capabilities to perform a behavior.

*Relatedness* includes processes that create a meaningful connection, convey understanding, and engage participants with the process [48]. In addition to actual persons participating or supporting the behavior change, the connection can be experienced with individuals who would benefit from the change (eg, getting fitter to be a better team member or focusing on one’s health to have more energy to spend with family). A digital intervention may also provide a sense of relatedness with the service, for instance, through experiencing the good intentions of the people providing the service. Relatedness to the intervention provider may be supported by taking the user’s perspective with empathy, displaying appreciation or concern, involving the person, gathering knowledge about the person and paying careful attention to them, dedicating time and energy, and being available when needed [50].

Smartphones provide many opportunities for supporting these needs, for instance, by offering accurate and usage-based or sensor-based feedback or by adding fun and motivating challenges with gamified elements [51-53].

### Gamification

An increasingly used approach for engaging users in the behavior change process is gamification, which refers to using elements from games, such as points, badges, visualizations, challenges, and surprises, in nongame contexts [54,55]. While motivational interviewing provides tools for motivational self-reflection and thus supports the active and conscious fulfillment of psychological needs, gamification can provide experiences of autonomy, competence, and relatedness by adding fun and excitement in the activities [52].

Evidence on gamification of behavior change interventions is sporadic because of varying multidisciplinary terminology and a lack of randomized controlled trials [56]. Some studies show promise in increasing user engagement [57,58], motivation [58,59], and physical activity [51,60,61]. Game mechanisms might also support the use of BCTs: apps with game elements have been found to use more BCTs than health apps in general [56]**.** Despite the promise, gamification is still used relatively rarely [56].

Game elements do not necessarily require users to reflect on their reasons for behavior, but they typically use intrinsic motivators, such as challenges and surprises, that may provide a spontaneous route to behavior change. For instance, users may engage in goal setting and self-monitoring of physical activity because of visualized goals and achievements instead of health targets. A successful example of this is an augmented reality game, Pokémon GO, which did not explicitly target physical activity but increased users’ daily steps with the game mechanisms [51].

### Theoretical Framework for Engaging App Users in the Behavior Change Process

**Figure 1:**
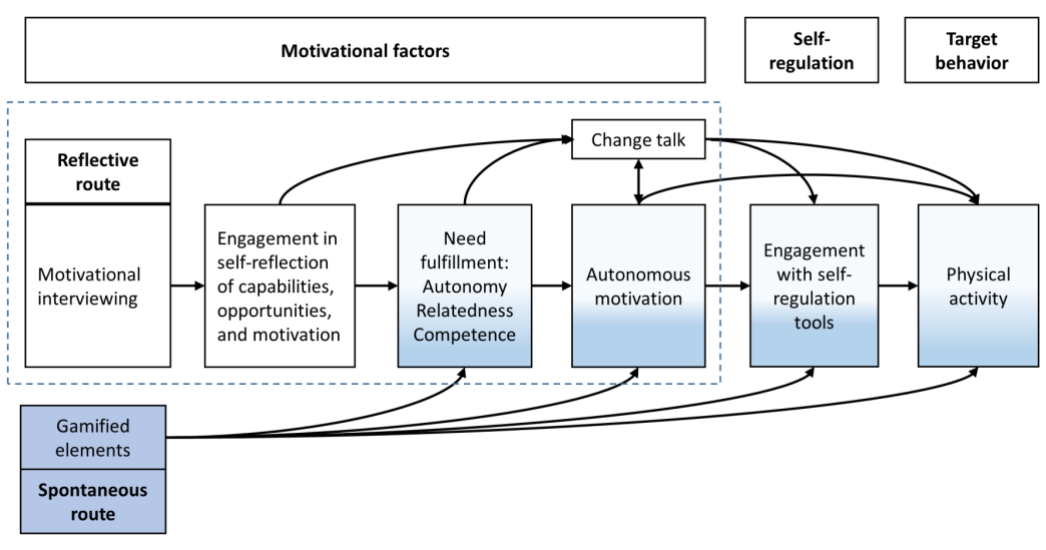
A proposed method for engaging users in the behavior change process. Digitalized motivational interviewing techniques help users engage in motivational self-reflection; identify reasons, capabilities, and opportunities for physical activity; and produce change talk. Gamification provides intrinsic pleasure with challenges and surprises. Together, these satisfy the self-determination theory’s psychological needs and support autonomous motivation. This increased awareness of the pleasure and benefits of exercise engages users in self-regulation, such as goal setting, planning, and self-monitoring, leading to increased physical activity. The dotted line shows the feasibility testing presented in this study.

To draw together the range of theories, approaches, and evidence surrounding engagement with apps for behavior change, we propose the framework depicted in Figure 1. Gamification provides intrinsic pleasure with challenges and surprises. Together, these satisfy the self-determination theory’s psychological needs and support autonomous motivation. This increased awareness of the pleasure and benefits of exercise engages users in self-regulation, such as goal setting, planning, and self-monitoring, leading to increased physical activity. The dotted line (Figure 1) shows the feasibility testing presented in this study.

We suggest that apps that support behavior change will be more effective if they engage users in the behavior change process. We characterize the behavior change process with the following three steps: (1) users are autonomously motivated to be physically active either by expecting intrinsic pleasure from the activity or by remembering that the activity supports their values, identity, or personally relevant goals [48]; (2) users enact self-regulation techniques, such as goal setting, planning, and self-monitoring [62]; and (3) users change their behavior (eg, walk to the supermarket instead of driving).

We suggest that users are more likely to actively engage in the behavior change process if the app addresses their basic psychological needs: supports their autonomy, creates a sense of relatedness, and provides them with experiences of competence and self-efficacy [42]. If these needs are met, users will have more motivation and other psychological resources to set challenging goals, make plans, and track their progress [45].

The psychological needs of autonomy, competence, and relatedness can be satisfied in two ways. *The reflective route* encourages users to actively consider their capabilities, opportunities, and the benefits of the behavior (eg, think what kind of physical activity they might enjoy or what they could achieve by being active) [63]. This self-reflection requires active cognitive engagement with the reflection tasks. Motivational interviewing is suggested as a method for engaging users in this motivational self-reflection because of its focus on building a successful working alliance and supporting engagement in behavior change [32]. Another pathway to engagement is through gamification. *The spontaneous route* does not require active self-reflection, as the game mechanisms can satisfy the psychological needs and engage the user in behavior change through intrinsic motivation [52]. This framework relying on reflective and spontaneous pathways draws from the studies by Hagger and Chatzisarantis and Strack and Deutsch [64,65].

### Aims of This Study

This paper aimed to present the development and feasibility testing of the physical activity–related components of the Precious smartphone app. The Methods section describes how the theoretical framework was employed in the Precious app: how theory and evidence were used to inform the selection of app features and how motivational interviewing and self-regulation techniques were implemented within the app.

Furthermore, to add to the emerging literature moving beyond quantitative usage metrics and describing how digital delivery affects the user experience, engagement, and intervention uptake [27,29,66], we have presented a feasibility study that examines user interactions with the app. Specifically, this feasibility study sought to understand the extent to which app use engaged users in active self-reflection.

Our hypothesis was that active cognitive engagement with the app content is necessary for effective commitment to the behavior change process. We studied user engagement with the motivational interviewing components focusing on two research questions (RQ):

RQ1: How did the users discuss autonomy support in the Precious app? This question aimed to understand how users approach autonomy support in smartphone apps and if interacting with the Precious app fulfills the psychological need for autonomy.

RQ2: What kind of change talk did the Precious app elicit in the users, if any? This question aimed to identify whether the motivational interviewing components met their target of engaging users in the behavior change process.

## Methods

### Service Design

The *PREventive Care Infrastructure based On Ubiquitous Sensing* (PRECIOUS) project consisted of eight European multidisciplinary partners in social and behavioral sciences, health psychology and psychiatry, computer science including human-computer interaction, nutritional science, and sensor engineering who worked together to create a digital platform supporting physical activity, healthy nutrition, sleep hygiene, and stress management. The final design of the Precious app was created through collaborative writing of project deliverables and weekly design meetings with social and behavioral scientists, computer scientists, and usability and graphic designers. The consortium reviewed relevant theory and evidence on behavioral sciences, gamification [67], socioeconomic factors, and business models as requested by the European Community’s Seventh Framework funding scheme [68] and created a system architecture for the overall service [69]. Each project partner identified the ethical and privacy principles related to their field of responsibility [70]. Full details on the project, including project publications, are reported [71].

The design of the physical activity arm of the Precious app started by drafting the theoretical framework (Figure 1), selecting the key service elements, and inventing ways to address the motivational and self-regulatory variables on a digital platform, through an iterative process of individual and collaborative design [72]. The systematic development of a motivational interviewing–based computer intervention by Friederichs et al [73,74] served as an inspiration for the motivational tools in the Precious app. The text content and algorithm of the user-specific tailored suggestions were written collaboratively by JN and KK and implemented by TG [75].

### The Precious App Development

The following sections describe the Precious app features and how these were drawn from behavioral theories. The aim of the Precious app is to increase users’ daily physical activity by supporting their commitment to the behavior change process: increasing their motivation, self-reflection, self-regulation, and physical activity (Figure 1). We used several techniques to reach this aim.

The Precious app addresses the following two aspects of behavior change: (1) motivating individuals who may not yet see the need for health-enhancing physical activity and (2) providing self-regulation techniques to help translate motivation into physical activity [45]. The Precious app’s tools for motivational self-reflection aim to increase autonomous motivation (eg, awareness of the personally meaningful outcomes of increasing daily physical activity). The self-reflection evoked by motivational interviewing and the personally relevant information on the effects of exercise from the biofeedback sensors aim to support active cognitive engagement with the behavior change process.

Increased motivation, such as joy of achieving challenges and salience of personally relevant outcomes, can help users to commit to the use of self-regulatory BCTs [45]. Goal setting, action planning, and self-monitoring will help users to initiate and sustain their activity. Altogether, the Precious app aims to remind users how their psychological needs can be met with physical activity. The functionalities of the Precious app are presented in detail in the following sections.

#### Relational Features

All texts in the Precious app aim to evoke relatedness with the app, drawing from the relational techniques of motivational interviewing and aiming to build an alliance with the user (Table 1, numbered with the taxonomy [33]). The motivational tools aim to create an encouraging environment open for exploration of options. They have been worded with the aim of acknowledging users’ efforts and self-worth (T1.2) and emphasizing their autonomy (T3.16). The messages aim to normalize possible motivation deficits and failures in reaching behavioral targets (T3.22). The tools paraphrase users’ selections and provide reflective feedback of the selections they had made previously (T1.3). All options are presented as acceptable and as a natural part of the behavior change process (T4.2). The app aims to transmit trust by assuming that users themselves know the options that are best for themselves (T4.5). All app content was written in empathetic and encouraging language, aiming to avoid any directive orders or judgmental feedback that could create guilt, shame, or feelings of being controlled, elements known to predict disengagement with activity [32,42]. These relational tools and self-reflection are hypothesized to satisfy the basic psychological needs of the self-determination theory and increase autonomous motivation [41]. Examples of the self-reflection tasks in each app are presented below.

Table 1 presents the ways motivational interviewing features target relational aspects and service engagement.

**Table 1:** Relational techniques from motivational interviewing implemented across the Precious app service (text in square brackets varies based on an individual’s previously indicated preferences or previously made choices).

Technique^a^	Description	Examples of implementation in the Precious app	Targeted psychological needs to increase service engagement
T1.1: open-ended questions	Questions that cannot be answered with a limited response (ie, yes, no, or rarely)	“Imagine yourself being active and enjoying it. How is your life different?”	Open-ended questions aim to guide the user to think of reasons to increase physical activity and the positive changes that it may cause. Guiding users to imagine also supports their autonomy to choose the activities they enjoy
T1.2: affirmations	Acknowledging users’ efforts and self-worth	“Well done! First app completed!” “Good job! You achieved your daily step goal.”	Acknowledging efforts aims to support the users’ competence and self-efficacy and create a sense of relatedness with the service. The users are hypothesized to return to the service, as they feel their efforts do not go unnoticed
T1.3: reflective statements	Paraphrasing users’ choices (from multiple choice answers to reflective feedback)	“OK. So, in other words, physical activity is important to you because it could help you to achieve your [top outcome goal].”	Paraphrasing aims to support self-reflection and provide perspective on the selections the user has made. It supports autonomy by valuing user-made choices and targets relatedness by providing an experience of being heard
T3.16: emphasize autonomy	Freedom to choose outcome goals, behavioral goals, and activities and their timing	“Earlier, you said that being physically active would help you to [achieve your top outcome goal]. Well, there are many different ways to be active, and some which you would enjoy more than others. Swipe forward to ensure you will get recommendations you like.”	This technique aims to support user autonomy and suggest activities that are intrinsically motivating
T3.22: normalizing	Acknowledging that it is not uncommon to find behavior change challenging	“Many people have difficulties recalling times when they enjoyed being active.”	Normalizing is used to nurture the sense of relatedness through empathy and compassion, even if users indicate no intention to be active
T4.2: consider change options	Neutral and supporting language to consider all options and no guidance to specific choices	“The whole point is to support you with things that matter to you most. The more you interact with Precious, the more accurate these recommendations will become.“	The neutral language used to support autonomy, competence, and relatedness, as users can feel that their choices are accepted and supported
T4.5: support change	Trusting users’ ability to choose best options for them and remind them of their choices	“Based on your responses it seems that you think a change in your [chosen behavioral target] can help you to [achieve your top outcome goal]. That's good to know! Precious will now help you on the path to getting more of what you want.”	Reminding users of their personally relevant goals may help them feel competent to execute their plans and autonomous to choose their goals and thus increase relatedness with the service

^a^Relational techniques from motivational interviewing as identified by Hardcastle et al [33].

#### Features Based on Self-Determination Theory

The Precious app’s design draws from the qualitatively different motivational styles of self-determination theory [48]. To address intrinsic motivation by adding pleasurable elements to physical activity, the app was built to contain gamified challenges and visualizations. Other aspects of *autonomous motivation* were targeted with tasks that evoke users’ personally important life goals and positive memories related to physical activity, aiming to remind them of factors that help them identify as an active person or that integrate physical activity as part of their lifestyle.

**Figure 2:**
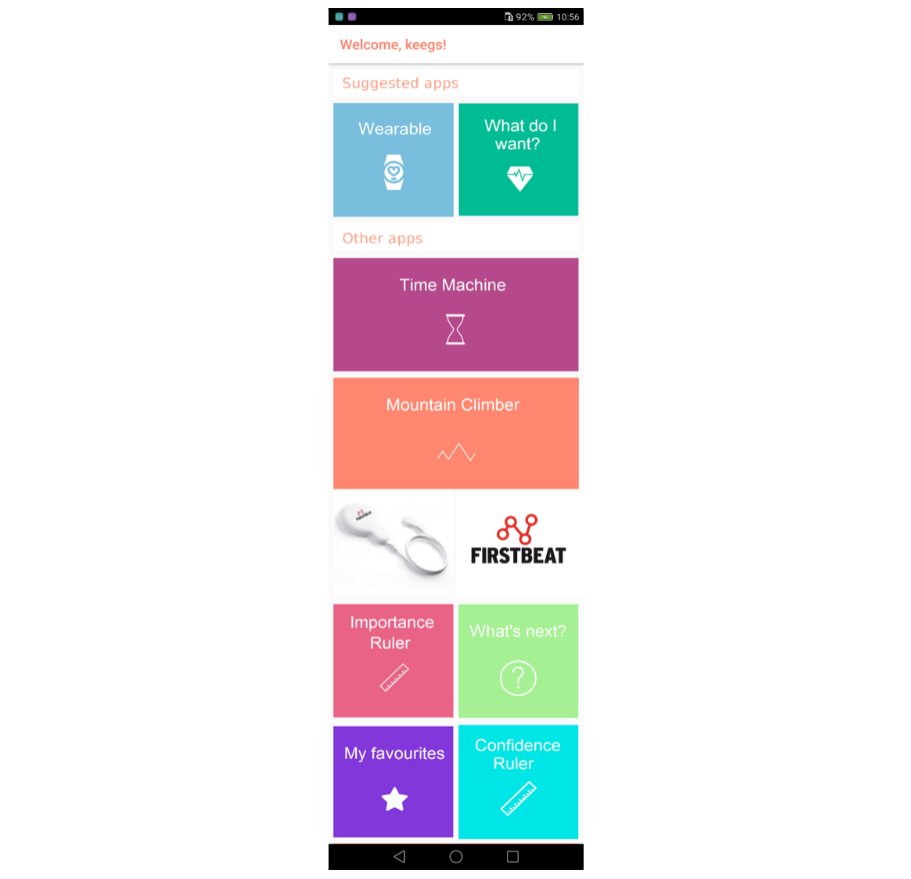
Implementation of the Precious app home screen, with suggested apps at the top of the screen.

Cross-cutting features in all app components are the psychological needs of the self-determination theory: autonomy, competence, and relatedness. To address the need for *autonomy*, the basic structure of the app was chosen to consist of freely available tiles (Figure 2). Each tile hides a specific app feature or BCT. On the basis of user selections, the service recommends certain tiles on the top of the screen, but all tiles remain available for the user to explore. Users are offered freedom to choose the values that guide them and the ways to achieve their outcome goals and to adjust their behavioral goal each day. The tiled structure also adds flexibility for further use of the platform, as software developers can add new tiles with new features without modifying the original content.

To offer experiences of *competence* and *self-efficacy*, the app notifies the user when approaching the daily step goal and celebrates achievements and service engagement with a notification. The self-regulation features in the Precious app are designed to increase competence by supporting individually tailored goals and comparing users’ results with their previous results only (weekly average), not with other users’ results. Instead of receiving a standard step goal, users are encouraged to set daily step goals that slightly exceed their current step average. Finally, users are encouraged to think about the good experiences they have had and might have with physical activity, hoping to remind them of times they have felt competent [75]. Competence is also addressed by aiming for an intuitive usability of the app functions, which has been associated with continued service use and higher experience of competence [76].

In addition to the relational techniques (Table 1), the need for *relatedness* is addressed with personalization. The main screen greets users with their name, and the motivational tools specify that recommendations the app provides will be based on their selections. To increase the relevance of the recommendations, algorithms of the Precious app suggest motivational elements to those who do not express interest in tracking or physical activity and self-regulation techniques to those ready to act.

Users proceed through the motivational tools by swiping the screen as if turning a page in a book. The rules engine of the Precious app was designed to approach the user after recognizing a period of inactivity and to ask whether the user’s previous choices still seem relevant or if they would want to reconsider their goals. This way the app is not imposing external requirements to the user but, with their permission, reminding them of their personally valued goals and activities [77].

#### What Do I Want?

**Figure 3:**
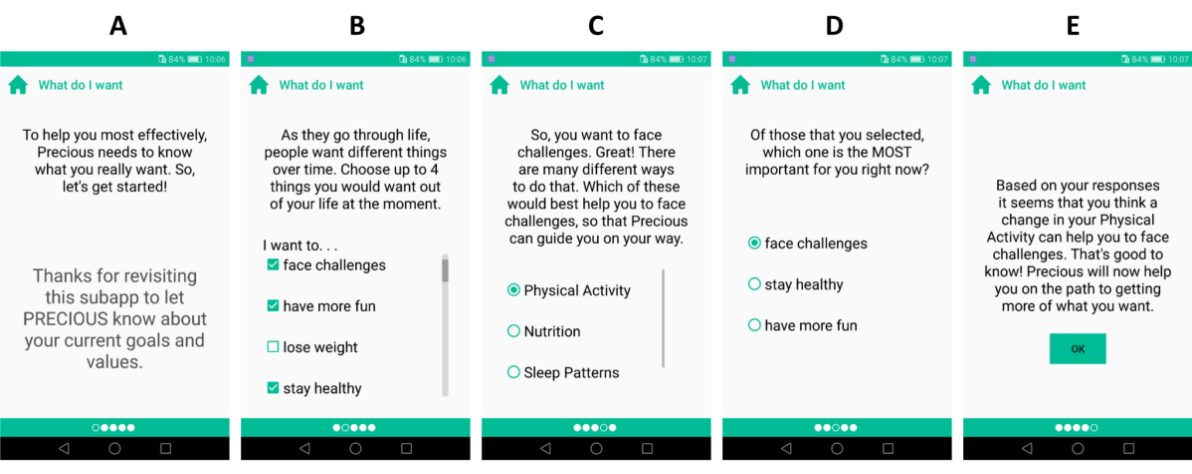
Implementation of the What Do I Want? tool. Screen B shows implementation of outcome goal selection, focusing on the things the user wants out of life, and Screen C allows users to indicate which of these are most important to them at this moment. Screen D provides a simple reflection on the content of the user’s chosen outcome goal and offers a menu of possible behavioral changes, which would be most likely to help them achieve the outcome goal set on Screen C. Screen E provides a summary of the user’s interaction with this tool, highlighting their chosen outcome goal and behavioral target.

The first motivational tool of the Precious app is called *What do I want?* (Figure 3). It builds on the values exploration in motivational interviewing and starts by suggesting a list of outcome goals (BCT 1.3, Table 2) [78]. To address individuals with low motivation for physical activity, we do not impose exercise- or health-related goals but also offer options such as *feel connected with other people*, *face challenges*, and *relieve stress or tension*. These outcome goals set the context for further interactions with the Precious app. This tool aims to increase autonomous forms of motivation by increasing the salience of personally valued goals. This is done by encouraging the reflection of desirable and beneficial things in life that may be achieved with physical activity. This tool uses relational features T1.3, T3.16, T4.2, and T4.5 (Table 1).

**Table 2:** Behavior change techniques in the Precious app.

Name of the feature		BCTs^a, b^		Description		Targeted behavior change mechanisms	
**Motivational components**							
	*What do I want?*		1.3 Outcome goal setting		The Precious app prompts users to reflect on their life goals and to choose their preferred outcome goal(s) from a list.		Selecting personally relevant outcome goals is hypothesized to support autonomy and nurture the relatedness with the service. Thinking about personally important reasons to be active may increase autonomous motivation for physical activity.
	*What do I want?*		1.7 Review outcome goals		If a user’s engagement or activity levels decrease, the Precious app asks them to consider selecting a new outcome goal in the *What do I want?* tool.		This technique aims to support user’s relatedness with the service by empathetic concern of the user and by acknowledging that the current support offered may not be optimal. Providing new outcome goals to choose from targets user autonomy. Together these aim to increase motivation for physical activity.
	*Time machine*		15.2 Mental rehearsal of successful performance		The Precious app asks users to visualize a future event in which they would enjoy physical activity and prompts users to reflect on the positive consequences of this experience.		Mental rehearsal provides full autonomy to choose the activity, the environment, and the company. This technique can thus address autonomy, competence, and relatedness and increase motivation to try out the activities in real life. The user may identify new or forgotten opportunities and capabilities for activity.
	*Time machine*		15.3 Focus on past success		The Precious app asks users to reflect on a past event in which they enjoyed physical activity and prompts users to reflect on the positive consequences of this experience.		This imagination technique is targeting the psychological needs of autonomy, competence, and self-efficacy, reminding users of the moments they enjoyed being active and thus boosting their motivation. If users have good exercise memories with other people, this technique may also remind them of the sense of relatedness. The user may identify forgotten opportunities and capabilities for activity.
	*How to get there?*		Additional BCT: linking behavioral goals with outcome goals		The Precious app reminds users that their behaviors (eg, football and gardening) can help them to achieve their outcome goals (eg, feeling connected to others and having fun)		This technique is expected to create a mental bond between users’ valued goals and the tangible actions that help them achieve those goals. As both goals and behaviors are self-selected, this technique targets all three psychological needs of autonomy, competence, self-efficacy, and relatedness and may thus lead to increased motivation. The tool may help identify such opportunities to be active that serve a purpose
	Smartphone notifications and biofeedback report		10.4 [Digital] Social reward		The Precious app delivers smartphone notifications with positive messages based on tracked service engagement or activity. Biofeedback reports include praise and encouragement for progress.		Supportive but accurate feedback aims to increase users’ competence, self-efficacy, and relatedness with the service, which again should increase motivation to take care of their well-being
**Self-regulation techniques**							
	*Mountain climber*		1.1 Behavioral goal setting		The Precious app allows users to set a daily step goal. To set a realistic goal, users see a suggestion of their past 7-day average as a starting point.		This self-regulation technique targets users’ autonomy by letting them adjust their daily goals. Basing goal recommendations on each user’s step average takes into account their capabilities and aims to increase competence and self-efficacy. Users can consider their opportunities to be active on the day while setting a goal.
	*Mountain climber*		1.4 Action planning		The Precious app allows users to plan bouts of physical activity, including activity type, intensity, and time of day. Users then receive notifications when their planned activity is approaching.		Users’ autonomy is supported as they can choose any physical activities and be supported in completing those. Making plans with the tool can remind users of their capabilities and opportunities for activity as they see the list of activities they like.
	*Mountain climber*		1.5 Review behavior goal(s)		When opening the Mountain climber tool, users can review their previously set goals, the extent to which those were achieved, and adjust the goal for the current day.		Seeing their past behavior visualized as mountain panorama and achievements as flags on top of the mountains may help to celebrate successful goal achievement, increasing competence, self-efficacy, and awareness of capability. Users can change the daily goal anytime, which targets autonomy.
	Smartphone notifications		1.6 Discrepancy between current behavior and goal		The Mountain climber tool shows in real time how many steps the user has taken and how far they are from their daily step goal. The user also receives messages on the percentage of steps that they have accomplished by afternoon.		Getting a reminder of goal progress may increase the sense of competence and self-efficacy in case users have already achieved their goal or they feel they can complete the remaining activity during the evening. Seeing the difference between their goal and current situation may create an intrinsically motivated challenge to achieve the goal.
	Smartphone notifications		2.2 Feedback on behavior		The Precious app sends a notification about progress toward user’s step goal and goal achievement.		Supportively worded messages about goal progress can increase relatedness to the service and sense of competence and self-efficacy if the goal seems achievable.
	Activity bracelet		2.3 Self-monitoring of behavior		The Mountain climber app displays the number of steps a user has accumulated each day. This step total aggregates steps logged by the activity bracelet, the phone’s onboard accelerometer, and manually logged activities. Users are asked to manually log other activities than walking, running, and cycling (which are automatically tracked). Action plans made with the tool can be marked completed with a single tap.		Aggregating activities from several sources can help users understand how all activity contributes to the daily total and that all occasions to be active count. This, in addition to visualization of their activity as a mountain panorama and achievements as flags on top of the mountains, may help to celebrate their efforts, increasing competence and self-efficacy. Seeing activities visualized as mountains to conquer may increase intrinsic motivation to use the tool.

^a^BCT: behavior change technique.

^b^Behavior change technique numbering based on the study by Michie et al [8].

#### Importance Ruler

**Figure 4:**
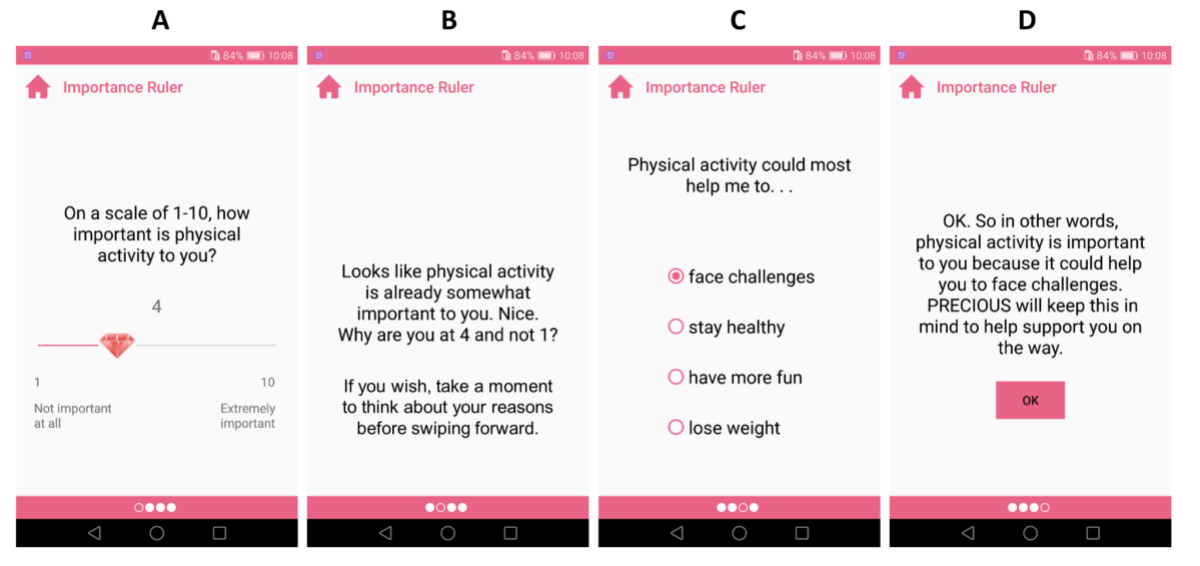
Implementation of the Importance ruler tool. Screen A shows the importance ruler itself. Screen B shows a follow-up question from the individual’s initial response and a prompt to help users better introspect about why they chose a particular number on Screen A. Screen C shows a selection of possible positive outcomes of physical activity, populated from the choices made previously in the What Do I Want? tool. Screen D shows a reflection of the user’s chosen responses on Screen C.

A technique taken directly from motivational interviewing, Importance ruler, first asks users how important they perceive physical activity on a scale of 1 to 10 (Figure 4) and then, depending on the answer, follows with an affirmation and asks why the user did not choose a lower number. The user is guided to think of reasons that would make physical activity matter to them. This reflection task is followed by a reminder of their previously chosen outcome goals. The purpose of this is to help the user to create a mental link between physical activity and their personally valued goals. Depending on user selections, this tool uses relational features T1.2, T1.3, T3.16, T3.22, T4.2, and T4.5 (Table 1).

#### What's Next?

**Figure 5:**
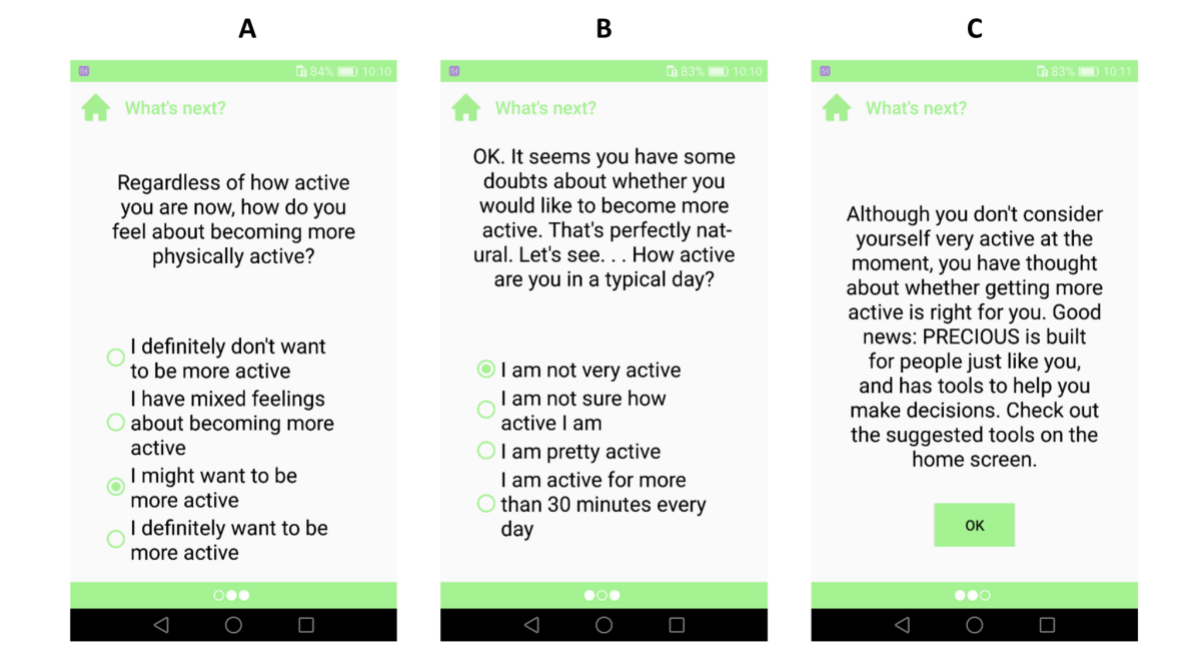
What’s Next tool. This tool assessed a user’s stage of change for physical activity and directed users to either motivational or self-regulatory features based on their responses.

This tool aimed to assess users’ motivation and activity status (stage of change [77]) to tailor the recommendations on the home screen (Figure 2) [75]. Indicating low motivation and intention for physical activity leads to features based on motivational interviewing and gamified challenges. If the user expresses intentions to start or maintain activity, the main screen will suggest goal setting, action planning, activity logging, and gamified challenges (Figure 5). Independent of user’s selections, this tool aims to acknowledge users’ efforts and self-worth by using empathetic language. This tool includes relational features T1.2, T1.3, T3.16, T3.22, T4.2, and T4.5 (Table 1).

#### My Favorites

**Figure 6:**
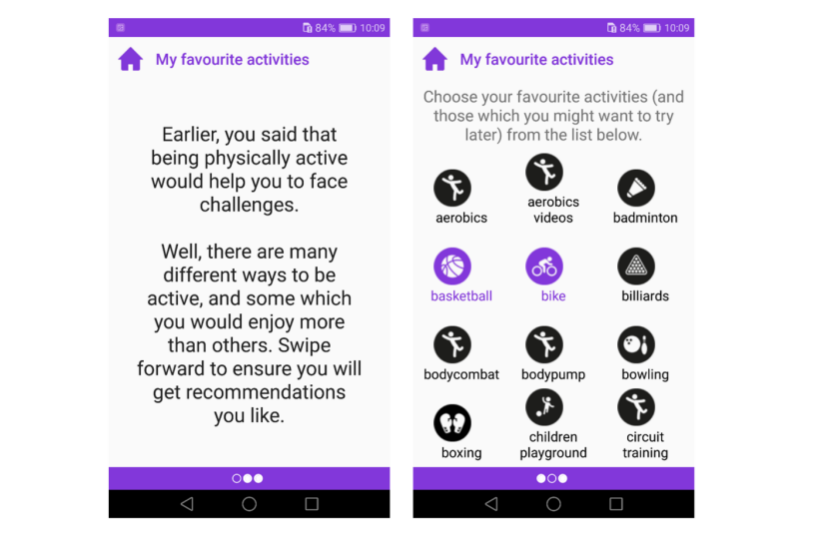
Implementation of the My Favorites tool, in which users choose the modes of PA that they might like to undertake. The prompt on Screen A recalls the outcome goal the user set in the What do I want? tool.

To acknowledge users’ efforts and self-worth, users are asked to choose physical activities that they have enjoyed in the past and/or might like to try in the future (Figure 6). This offers them a freedom of choice, may evoke positive memories of exercising while going through the list of options, may present opportunities for activities that the user was not thinking as physical activity (eg, gardening), and populates the database with options that may be used for later reminders, thus offering an experience of personalization. The chosen activities are also shown as the first suggestions in the self-regulation tool to improve the user experience. The *My favorites* tool was implemented with a simple tiled structure that shows all activities simultaneously and allows adding and removing them with one touch. The app was not finalized by the time of feasibility testing. The *My favorites* tool uses relational features T1.2, T3.16, T4.2, and T4.5 (Table 1).

#### Confidence Ruler

**Figure 7:**
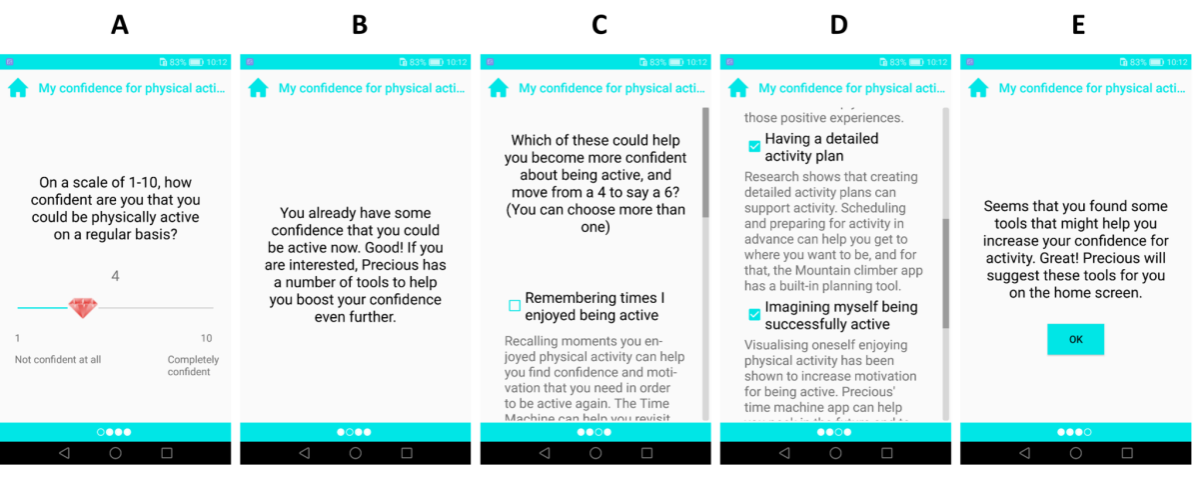
Implementation of the Confidence Ruler tool. Screen A shows the main question of this tool, which is followed up with a tailored reflection on Screen B. Screens C and D show a number of the tools available to users and the rationale for how each could help improve their confidence. Users were free to select as many or as few of these as they wished.

The *Confidence ruler* tool implements a core technique of motivational interviewing and starts with the question “How confident are you that you could be physically active on a regular basis?” It then provides feedback on user choices and leads to asking which of the app’s available tools could help the user to be more confident in their ability to be physically active. The answer options consist of all the tools that the Precious app has to offer and a rationale for their use (Figure 7). This tool aims to acknowledge users’ efforts and self-worth (T1.2) and, depending on the user’s selections, uses relational features T1.2, T1.3, T3.16, T3.22, T4.2, and T4.5 (Table 1).

#### Time Machine

**Figure 8:**
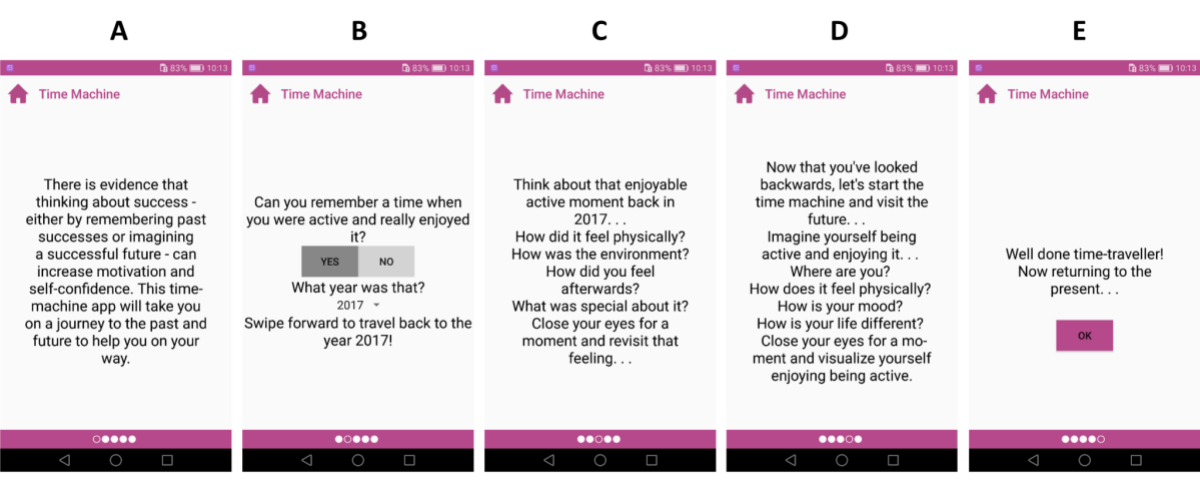
Implementation of the Time Machine tool. Looking back exercises are shown on Screens B and C, and looking forward exercises are shown on Screen D.

The *Time machine* tool aims to boost motivation, a sense of competence, and self-efficacy by evoking users’ positive exercise memories and helping them to create vivid images of successfully engaging in physical activity and enjoying it (BCT15.2 and BCT15.3, Table 2). A mental rehearsal of a successful performance was found to be the most effective BCT in increasing intention to be physically active in a recent meta-analysis [21]. To create a gamified, *machine-like* experience, the app first asks whether a user has any positive experiences and then the year the user wants to be sent to. This is followed with detailed questions about their experience that aim to acknowledge users’ efforts and self-worth (Figure 8). This tool uses relational features T1.2, T3.16, and T4.2 (Table 1).

#### How to Get There?

The aim of this tool is to strengthen the mental link between users’ outcome goals and the specific actions that help them achieve it. This tool is suggested after completing the values exploration in *What do I want*? and selecting favorite physical activities in *My favorites*. The task is to select those activities that take the users closer to their outcome goal. For instance, if the user has chosen the outcome goal *feel socially connected*, the next step is to scroll through their favorite activities and choose the ones that allow them to connect socially (Multimedia Appendix 1). This feature helps the users to build a personal rationale for doing physical activity. This tool was not finalized by the feasibility testing.

#### Biofeedback

**Figure 9:**
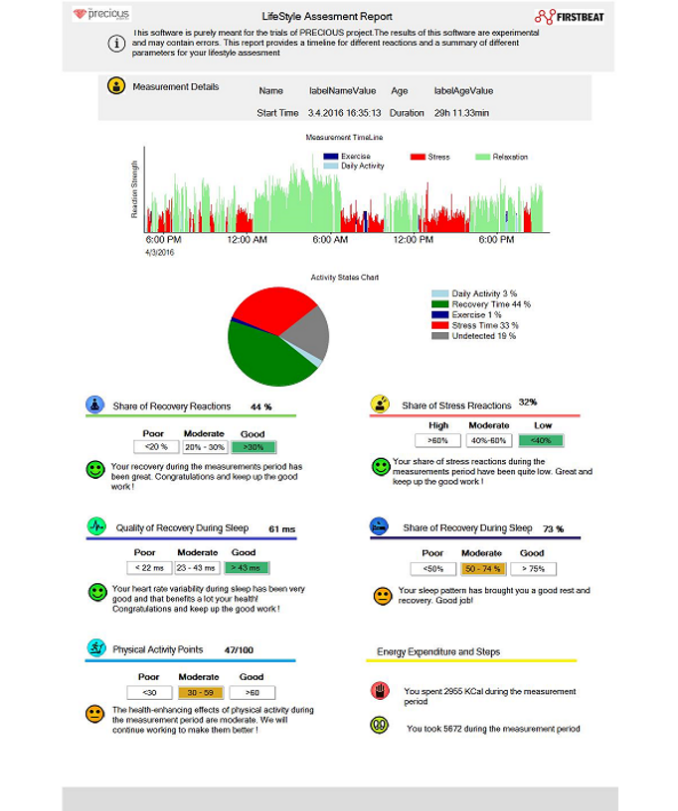
Example view of Firstbeat heart rate variability report for Precious.

We integrated Firstbeat’s (Firstbeat Technologies) heart rate variability sensor, Bodyguard 2, to the Precious app to offer the users the possibility to receive feedback on their physiological stress, recovery, and sleep quality (Figure 9) [79]. The feedback consists of graphs showing users’ activity levels in colored bars and interpretations of these bars telling the user whether their levels of activity and recovery have been health enhancing. Encouraging messages congratulate the users or support them to keep up their efforts. Users can access the reports in the Precious app behind a tile picturing the Firstbeat sensor after downloading the sensor data to the internet via a computer with a USB port. The aim of the biofeedback tool is to remind users that their behaviors have both immediate and long-term consequences on their well-being. Strengthening this mental link between their behavior and desired outcomes could strengthen their autonomous motivation. The feedback aims to acknowledge users’ efforts and self-worth by congratulating them for physical activity, good sleep quality, and recovery. The biofeedback report uses relational features T1.2, T3.22, and T4.5 (Table 1).

#### Gamified Elements

In addition to the gamified features of the Mountain Climber self-regulation tool, other components were also envisaged to help foster intrinsic motivation for using the Precious app [52]. These components were all built into working prototypes but only after the completion of feasibility tests that were undertaken with the app components described earlier.

#### The Journey

Swiping left in the main menu reveals the user their journey, a map of achievements. Completing the motivational tasks and activity challenges creates a badge on a background of changing landscapes. All actions accumulate points, with less points obtained from app use and more points, from sustained streaks in physical activity. Journey’s main function is to increase the sense of competence and self-efficacy by showing visible cues of progress and listing all the actions taken on the way to an active life. The changing landscape may motivate users to complete actions to progress into new levels (Multimedia Appendix 2).

#### Conquer the City

**Figure 10:**
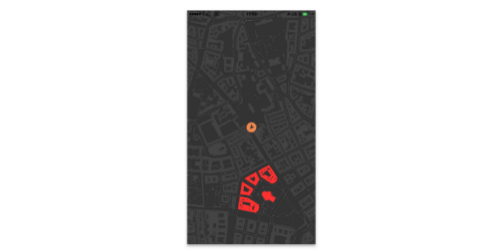
Conquer the city, location-based activity game.

A location-based activity game was designed to create entertaining challenges, tasks, and competition that would make walking intrinsically enjoyable. The aim of the game is to conquer areas on the map by walking around them and by collaborating and competing with other app users. This addresses the psychological needs of relatedness and adds an element of excitement and fun for those who enjoy competition [80,81]. Users could also challenge themselves, trying to conquer new areas or specific targets on the map (Figure 10).

#### The Precious App Development: Self-Regulatory Tools

The purpose of the self-regulation tool is to give users a clear picture of their current activity level and how that compares with their target level (self-monitoring, BCT 2.3; discrepancy between current behavior and goal, BCT 1.6, Table 2 [8]) and guide them to set daily, realistic, and achievable activity goals (BCT 1.1). The planning tool was created for creating detailed activity plans (BCT 1.4) and logging of activities that were performed while not wearing an activity bracelet (BCT 2.3). To make these techniques intuitively usable, we created a graphical interface that aggregates all the activity the users have accumulated during the day, their daily goal achievement, and their progress over time.

#### Mountain Climber Self-Regulation Tool

**Figure 11:**
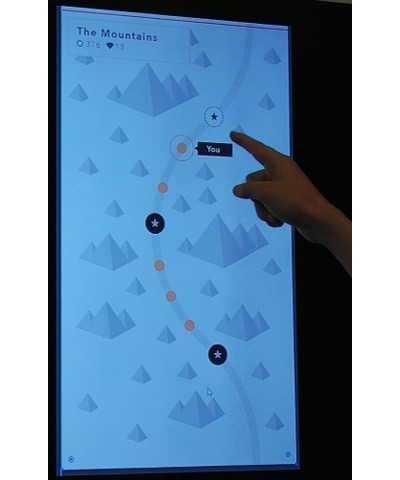
Implementation of the Mountain Climber self-regulatory tool. Screen A shows the main view, with blue mountain indicating an achieved goal. (Green tags on Screen A were not visible to users and are included here to indicate the destination screen after a tap action.) Screen B shows the step goal setting function. Screen C shows the action planning function. Action planning and logging are done by choosing an activity from a dropdown menu and setting the start and end time. The user can also adjust the intensity of the activity to either low, medium, or high, the default being medium. For ease of use, action planning is done in the same way as activity logging, with only the time being set in the future. The visual symbol of the planned activity appears in dim grey color, and the steps contribute to the daily total only after the user touches the button “I did it!”. Screens D and E show a detailed daily view, with planned activities in grey and completed activities in orange, indicating how many percentages of the daily goal is reached with the activity.

These requirements lead to the development of *Mountain climber* (Figure 11), designed with game-like features, with the aim of increasing user engagement in self-regulation. These include the graphical presentation of the accumulated steps (mountain) and visual rewards for goal achievement (illustrations of mountain life) that appear randomly after successful goal achievement (by the time of the feasibility testing, only a flag on top of the mountain and a changing color were implemented). Mountain was considered a representation of daily achievement that would be easily understandable for every user and that would add symbolic meaning to the accumulating steps. Goal setting was done by scrolling a digital button on the screen and choosing a certain step goal for the day. The goal setting tool was set to display the mean of the past 7 days’ steps when opening the Mountain climber. This was designed to educate users of their typical step amount to encourage reflection of how much activity could be embedded in the day ahead. Late afternoon, users receive feedback in a message providing the percentage of the daily step goal they have achieved to adapt their evening activities and accumulate the missing steps. Consecutive usage days create a panoramic view of user’s physical activity, showing possible increases or decreases over time and reminding them of their achievements.

### Physical Activity Measurement

Within the Precious app, physical activity data are accumulated from three separate sources, which the app aggregates to a single step count for each day. Steps per day were chosen as the common metric for presenting physical activity data, as it is readily interpretable and allows for direct comparisons from day to day within an individual. To address the socioeconomic challenges related to the use of digital services [82], the Precious app was built as a stand-alone smartphone app, signifying that anyone with an affordable smartphone could use the service without accessories [83]. For increased physical activity data accuracy, the app can also be connected to external monitoring devices through Bluetooth. For pilot testing of the Precious app, the primary source of physical activity data was a wrist-worn activity bracelet (Xiaomi Mi Band 1S Pulse Bluetooth 4.0 IP67 Waterproof Smart Bracelet) [84]. Step counts from the activity bracelet were passed to the app via low-energy Bluetooth at regular 10-min intervals when a connection with the app could be established.

As users of the Precious app may not always wear their activity bracelet, and as some activities (eg, cycling and weightlifting) are not accurately recorded by wrist-worn accelerometers, users can also manually log their activities. The Mountain climber tool allows users to select a physical activity from a list and to specify the time and intensity of this activity. Knowing a user’s height and weight, the Precious app converts the activity to steps using metabolic equivalent (MET) values and the following equation [85,86]:

Steps/minute for activity = (MET value of activity ×112.5)/3.5

As a final source of physical activity data, the Precious app used data collected from the phone’s onboard accelerometer and the Google Play Services Fitness application programming interface (API). This allowed for identification of steps taken as well as minutes spent walking, running, cycling, traveling in a vehicle or being sedentary. This source of physical activity data was used in periods where the app was unable to communicate with the activity bracelet, and no physical activities had been manually entered.

When amalgamating data from various sources, users’ manually entered activities were given the highest priority. Any step counts received from the Mi Band or onboard accelerometer that had timestamps that overlapped with the timestamps of manually entered activities were not added to an individual’s total. Steps obtained from the Mi Band were given the second highest priority. Steps obtained from Google Play Services Fitness API were given the lowest priority and were only added to an individual’s total in periods in which the Mi Band had not recorded any new step count activity. The aggregation of these three data sources was presented to users in real time, which offers users a comprehensive and realistic picture of how their steps accumulate during the day, how different activities contribute to the whole activity, and how their activity varies daily.

### Feasibility Testing

#### Participants

The interviewers recruited a convenience sample of 12 adults living in two different Finnish regions, with diverse educational backgrounds (three without any university studies and one with a doctoral degree) and ages ranging from 25 to 63 years. Of 12 participants, 8 were women and 4 were men, both genders ranging from physically inactive to highly active. Participants had different levels of experience with smartphone apps, ranging from active sport app users to participants without a smartphone. With think-aloud studies, five participants are deemed sufficient for detecting most usability problems [87,88]. To assess the feasibility of the digitalized motivational features, we recruited the maximum number of participants who could be interviewed within the project time limits, increasing the participant number to 12.

Overall, two participants (P6 and P7) agreed to participate only in a think-aloud study of the biofeedback report and were thus excluded from the current analyses (participant details are in Multimedia Appendix 3). The Precious app study received a favorable decision from the University of Helsinki Ethical Review Board in the Humanities and Social and Behavioural Sciences in January 2016. The project also addressed ethics and privacy in yearly reports to the European Commission. Participants were interviewed in person by a research group member (PhD researcher or intern) in June 2016 at a location that was convenient for them (office, home, or university lobby) and were rewarded with a movie ticket. Inclusion criteria were written informed consent, aged at least 18 years, sufficient language skills to use the English language app, and to conduct the interview in Finnish or English.

#### Procedure

After providing informed consent, participants were provided with a smartphone with the Precious app and a screen recording program, AZ Screen Recorder, and were asked to practice the think-aloud method with the smartphone’s other preinstalled apps (eg, alarm clock and address book) until they were speaking continuously, describing everything they saw, thought, or did with the app [89]. Participants were then instructed to use the Precious app just as they would any other app they had just downloaded and to think aloud while doing so [89]. Participants were also requested to ask any questions that came to mind as they explored the app (eg, where they should tap or what does a certain button do), as such questions could help us better understand user experiences [89]. Participants could freely interact with the Precious app until all content had been explored (usually around 30 min, including the dietary features not presented in this study), and during this time, if a participant stopped thinking aloud or passed several features without commenting on them, the interviewer prompted them by asking what they were thinking. After the think-aloud study, participants were briefly interviewed about their physical activity levels and previous experiences with smartphone apps.

### Measures

To assess participants’ physical activity levels, we used a validated single-item question, “On a typical week, on how many days do you do a total of 30 minutes or more of physical activity, which was enough to raise your breathing rate. This may include sport, exercise, and brisk walking or cycling for recreation or to get to and from places but should not include housework or physical activity that may be part of your job” [90].

Think-aloud walkthrough interviews were followed by a semistructured interview with open-ended questions on participants’ previous experience with mobile apps, general perceptions of the Precious app and its most/least useful features, suggestions for improvement and usage, possible features that could exclude potential users, and factors related to engagement with the service. These questions were mainly used for the technical usability analyses not presented in this study. Interviews and think-aloud walk-throughs as well as participants’ on-screen interactions with the app were video recorded. Interview recordings were transcribed verbatim by a research assistant and a trainee. User actions such as tapping and swiping the screen were also transcribed, as the analysis of user engagement included all interactions with the app.

### Analysis

Transcripts for each participant from think-aloud and semistructured interviews were analyzed with a deductive, theory-based thematic analysis, informed by Braun and Clarke’s phases of thematic analysis [91], used previously to analyze think-aloud studies of smartphone use [92,93]. The method allows for the essential content of the interviews to be captured in themes that describe the data patterns in a summarized form [91]. First, we analyzed data from each participant individually to remain sensitive to their experience and to detect possible differing themes between participants. Second, themes were synthesized from each participant into a general set of themes. Discussions between three researchers (JN, KK, and AH) led to agreement on the primary themes.

The theory-driven RQs on change talk (motivational interviewing) and autonomy support (self-determination theory) were analyzed with a deductive approach, aiming to identify any passages that fit these theoretically defined constructs [91]. Change talk and sustain talk (counter-change talk) were coded following the guidelines of the CLEAR (Client Language EAsy Rating) coding system [94]. Following the CLEAR guidelines, any factual information of existing behavior was not coded as change talk (eg, “I walk to work every day”), and choices made at the ruler tasks were coded as change talk only if there were confirmatory comments [95]. The excerpts on change talk and autonomy support were analyzed with their semantic meaning, assuming a unidirectional relationship between meaning and experience and language [91]. After identifying all the change talk–related passages, these excerpts were then coded as specific subtypes of change talk (ie, theory-driven themes) [91]. Finally, to optimize the usefulness of the results for the reader, all data excerpts on change talk, marked with the theme they represented, were organized and summarized under the respective app functions. Presenting change talk under specific app functions allows the reader to evaluate the tools and techniques that can be reproduced elsewhere, as suggested by O’Halloran et al [36].

## Results

### Feasibility

The feasibility analysis on participants’ engagement with the Precious app answered the following two RQs: (1) how participants discussed autonomy support during the think-aloud walkthrough and the end interview and (2) what kind of change talk did the Precious app elicit in the users during the walk-throughs.

### Research Question 1: Perceived Autonomy Support During Interactions With the Precious App

We identified the following themes around the original, theory-based theme *autonomy support*: valuing the chance to choose, autonomy supportive feedback, expecting controlling features, concern about lack of autonomy, and autonomous goals.

#### Valuing the Chance to Choose

Consistent with the self-determination theory, participants valued autonomy supportive features, especially the chance to personalize the content and make selections:

It is good that this background information is added there so that it’s not like the same for everyone. Absolutely, it’s good to have it, so that you can influence it yourself.

P8

The chance to choose was especially relevant for the self-regulation features, as participants appreciated the possibility to adjust the daily step goal and consider the day ahead:

So, every day you can set the goal. But that sounds smart. Maybe a bit smarter than what I have in use, where there’s for every day that...I think it has 10 000 the...Or you can’t set a goal, they think that 10 000 is the recommendation...I think this is smarter that you can also...if, like, you know that this day will be...that maybe there’s not so much walking, so...

P4

A participant with low levels of activity thought she would take advantage of the ability to set personal step goals to make sure she would achieve them:

I guess I’d probably put, if you could put, beforehand the goals, so, I’d probably put so… maybe even lower? So that they would be really, like, achievable and probably I would achieve them.

P11

The number of personalization options was generally found to be reasonable, and users found options that pleased them. However, overall, two participants felt that the list of 20 outcome goals had too many options to choose from:

I: What are you thinking?

P9: Just that there’s a lot of options again, there was four a moment ago and now there’s like forty...already reading these, now that it’s a busy situation...well at least, I’ll tick some of these.

P5: [chooses Feel more healthy] Yeah, there were too many. And again, you can choose so many different ones.

When asked if the service would exclude any user groups, one participant saw the number of options as too high for elderly people:

Then if the idea was that this is for elderly people, then it might be a problem that there’s so much of everything. This might be like a shock, or at least I remember when I’ve tried to teach my grand-parents Internet and computer use, and just when you open the screen and there’s a lot of small icons, it’s often very difficult. And when you need to click and...really self-evident stuff can become a threshold, but I don’t know for how difficult population you are aiming this.

P10

#### Autonomy Supportive Feedback

In addition to freedom of choice and tailoring, participants wished to receive encouragement and praise and mentioned that the tone of interaction is important:

Although [another sport app] it’s just an app, but it says something like “now you’ve missed your training session,” it makes me feel somehow bad. So probably you should pay attention to that, how the feedback is.

P4

#### Expecting Controlling Features

The interviews revealed that participants were used to apps that prescribed specific goals or activities. They assumed that the app would tell them, for instance, how much activity they are expected to do:

P12: Here it’s like 36 percent. 36% of what? Like...

I: Yeah, that’s not clear?

P12:

Taps 36% circle. Nothing happens

P12: Probably like how much should I walk or something.

These expectations were based on their previous experiences with apps that provided less autonomy support:

This is slightly different than what I’ve previously used of these health apps. Usually, very first question is height and weight and target weight. That’s the most common. But well, this seems good...or like good so that it’s not always necessarily the weight loss. That usually those apps have weight loss as default...

P4

#### Concern About Lack of Autonomy

In addition to expecting health apps to be controlling, participants also expressed concern about this *lack of autonomy*, for instance, that their early choices would tunnel them into unwanted recommendations later:

And then, choosing “stay healthy” brings to my mind immediately that now [Precious] will suggest to “take long walks” and “do yoga” and all these healthy activities, while I don’t like that at all. So, it instantly brings to my mind those stereotypes that this kind of healthy activity includes. Which you might not want yourself- although I choose this “Stay healthy,” I think however if I should have put some “Face challenges” so that I will get some fun to the activity.

P9

How is it programmed then [to provide suggestions]...Does it affect a lot if you choose the wrong one of the two

goals]...[P8

One participant experienced the question about his intention to be active as expectations set on him (*What’s next* tool) as expectations set on him rather than being tailored:

This here thinks I’d need to change the level of physical activity, I personally thought about it more like...that it’s just one central aspect...I didn’t see I would somehow need to change it.

P10

#### Autonomous Goals

Most users made selections that could be identified as autonomously motivated outcome goals, wanting to achieve health, well-being, fun, and challenges with physical activity instead of aiming for external goals. Several participants reflected about the importance of different types of goals:

P9: I am very stiff, I should probably put that [“feel more flexible”] but I don’t really care about that I am stiff.

Chooses “Increase my stamina”

I: So, you took into account that it asks what you want

P9: Yeah...Probably it would be good to be a bit more flexible...And I should lose some weight but that’s not such a top thing for me...Would it be good to choose four?

I: Yeah, is it unclear whether you need to choose?

P9: No, it isn’t, no you don’t need to but...Well, there are that kind of options that I could choose from.

Chooses the option “Relieve stress or tension”

P9: Health is the most important.

Chooses the options “Maintain my functional ability” and “Stay healthy”

### Research Question 2: Change Talk Elicited During Interactions With the Precious App

We examined whether and how app usage encourages self-reflection by observing the occurrence of change talk. Within the theoretical framework of motivational interviewing and the original theme of change talk, we identified the following themes: desire, need, reason, ability, commitment, taking steps toward change, sustain talk, and ambivalence. With this method, we aimed to analyze the feasibility of digitalized motivational interviewing features, and not users’ views on behavior change. Thus, to support replication and further development of digitalized motivational interviewing, the need identified in a systematic review [37], examples of the themes are presented under specific tools.

#### What Do I Want? Tool

The *What do I want?* tool prompts users to select outcome goals that matter to them and to select behaviors that help them approach those goals. During think-aloud walk-throughs, all participants actively engaged in selecting options that were relevant to them and most of them expressed some form of change talk. The change talk produced was mainly of the *desire* and *reason* type [32], as, implicitly, these occurred each time a participant made a selection of the *things that they would want out of life*:

Hmm [reads options] maybe, maybe, maybe not, yeah quite nice. Well maybe, I want at least to be stronger. (Change talk—desire)

P2

The list of outcome goals seemed to help some participants to find *reasons* for activity. A quote from participant 10 shows how he actively reflects what the options would mean in his life, first rejecting several options, but then finding suitable goals:

Do I want to “face challenges.” Well I don’t know what these challenges mean here so it is a bit...maybe it has something to do with sports then. Not in general, I don’t have that feeling that I would like to face challenges...“More fun...,” I am quite satisfied with my level of fun at the moment, and I don’t feel that I would need to lose weight either, and staying healthy isn’t...well, it is good, of course. Let’s put that [selects “Stay healthy”]. “Functional abilities,” that is also a good thing. [Selects “Maintain my functional ability”]. (Change talk—reason)

P10

Participants also actively expressed *need* type of change talk, mainly when reflecting on which outcome goal options would be most relevant for them:

P9: [selects “Improve my general mood”] This is just what I need, to get out for a run or to be alone. - -

P9: Health is the most important. [Chooses the option “Maintain my functional ability” and “Stay healthy”] [chooses Physical activity as the behavioral strategy]. (Change talk—need, reason)

Although all participants engaged in the selection process and found four options that were suitable for them, not all of them actively voiced aloud the rationales behind their choices. Their *talk* consisted partly of the selections they made. We call this implicit change talk:

[Opens What do I want? tool] Hmm. So, I can put only one or then maximum four options here. [Selects “become more flexible” as an outcome goal] Okay, this is good. I become less...okay that’s it. Hmm. (Implicit change talk—reason)

P12

Presenting possible outcome goals as a list may not only be a positive resource for users. A couple of participants voiced sustain talk when arguing against a decision to choose certain outcome goals:

P9: I am very stiff, I should probably put that [“feel more flexible”] but I don’t really care about being stiff. (sustain talk—reason not to change) [Chooses “Increase my stamina”] (Implicit change talk—desire)

I: So, you took into account that it asks what you want

P9: Yeah...Probably it would be good to be a bit more flexible...And I should lose some weight but that’s not such a top thing for me...(need change talk and ambivalence)

Would it be good to choose four?

I: Yeah, is it unclear whether you need to choose?

P9: No, it isn’t, no you don’t need to but...Well, there are that kind of options that I could choose from.

Chooses the option “Relieve stress or tension”

P9: Health is the most important. [Chooses the options “Maintain my functional ability” and “Stay healthy”]. (Change talk—need)

Participants reflected their actual *needs* in relation to their current life situation:

The program asks, which of these [outcome goals] is most important, but it’s hard to give weight to one or the other.. On different moments, different things are important, but it asks what would be most important right now...Removing stress [selects “Relieve stress or tension”]. (Change talk—need).

P3

Sometimes participants did not express change talk spontaneously, but they appeared to have thought about the responses when asked:

P3: So, let’s see what is in there...So the program wants to find out what I really want, and...now I’m thinking what it asked.

I: So, what are you thinking?

P3: So, sharing happy moments, adventures, being in the nature, doing successful things. (Change talk—desire).

#### Importance Ruler Tool

All participants were happy to select a number describing their perception of the importance of physical activity, but as the app did not require text input, they swiped through the pages at a relatively fast pace. Only a few participants reflected aloud their choices spontaneously, but almost all provided reasons for the importance of physical activity when asked what they were thinking. We identified the themes desire, need, reason, and sustain talk from interactions with the importance ruler:

P1:

moves the pointer from 7 to 4

I: So, tell now, like, what you think or see?

P1:

moves the pointer from 4 to 7 and from 7 to 8

P1: I think that [physical activity] is important

I: Mm?

P1: Do I need to think about something else? [swipes forward] I guess not. I’ve been thinking for a second.

swipes forward

P1: Because of course I wanted to be more active. [swipes to the next screen, sees the preselected option ’relieve stress or tension’]. Yeah, that’s alright. (Change talk, desire)

P3: That it [physical activity] just keeps me active. It makes me feel healthy. So, in this question “physical activity would most help me to...” I would answer the button that was already selected, that it relieves stress and tension. (Change talk—need)

P9: So, I would want to choose immediately all of them [four user’s favorite outcome goals displayed], maybe least this “have fun”? Although it’s [physical activity is] very important to me and it’s fun for me.

But still, I’d see that if you need to think of health, then all these three others. (Change talk—reason)

Some participants chose the number by comparing themselves to their peers. Participant 9 had observed how the lack of activity has a different impact on her than on others:

Well...I don’t know, I just find [physical activity] fun. It is important to me to stay in good shape so that I have energy to do things in life and so on. (change talk—need)Also, maybe I compare myself to other people. Like, how important [physical activity] is to me compared to my pals. They don’t care if they can’t go for a run every now and then or if they move, but I become a bag of nerves. It starts to feel like you get mad from everything (change talk—need) so, that kind of thoughts.

P9

Participant 8 saw himself as a less active person than his wife, which affected his selections and elicited some *sustain talk*:

When I think of my wife when she always says that she will feel bad if she can’t go for a run, well, I am not at all like that. (sustain talk—reason not to change)

P8

#### Confidence Ruler Tool

The *Confidence ruler* tool asks about participants’ confidence to be physically active and offers options to increase the confidence (Figure 7). It was tested by only two participants because of late implementation. Both reacted in a way corresponding to their physical activity status: the highly active participant was feeling confident and expressed *ability* to change:

[reading the question on the screen] How confident am I with physical activity...Well, let’s say that I’m quite sure I could be regularly [active], let’s put for instance 9. (Change talk—ability)

P10

An inactive participant expressed a *need* to increase activity:

I guess...I am not that active, so I feel like there’s much to improve. (Change talk—need)

P11

While exploring the suggested BCTs in the *Confidence ruler*, participant 10 mentioned that a detailed plan might help him increase physical activity.

Ok, now this asks what would help me in this...Again, this suggests remembering...So, it’s kind of asking me these, ok...I wonder if it has automatically chosen me that one or if I have accidentally touched it myself. Some accurate plan could maybe [help me to increase physical activity] [selects the option Having a detailed activity plan] (Change talk—ability).

P10

#### What’s Next? Tool

The *What’s next?* Tool assesses users’ current levels of physical activity and intentions to be active to tailor further suggestions. The two participants testing the tool actively engaged, making the selections that were relevant for them. An inactive user expressed the *desire* to increase activity:

[reads the question “Regardless of how active you are now, how do you feel about becoming more physically active?”] Well I absolutely would want! [selects “I definitely want to be more active”]. (Change talk—desire)

P11

A physically active user did not see a need to increase his activity levels and, understandably, expressed sustain talk and ambivalence:

This here thinks I’d need to change the level of physical activity, I personally thought about it more like...that it’s just one central aspect...I didn’t see I would somehow need to change it. (Sustain talk—reason not to change)...Let’s say that I have an ambivalent feeling, do I want to be more physically active. (Ambivalence)

P10

#### Time Machine Tool

The Time machine tool suggested imagination tasks of pleasurable physical activity in the past or in the future. It was tested by only two users, and no change talk was coded from their interviews.

#### Mountain Climber Self-Regulation Tool

As opposed to the *needs-* and *reasons*-based change talk while using the motivational interviewing features, the change talk produced by the Mountain climber tool was *commitment* to the behavior change process and *taking steps toward change*. Participants used different Precious app tools in a random order, so this difference does not reflect the time spent using the app.

Participants saw the logging tool as a learning instrument and logging future activities as a commitment that might help them reach their goals. Participant 11 describes how she would learn about her activity levels while using the logging tool and could then increase her step goals:

Yes I believe I could even go and do [the planning], and I find it nice that I could add the goals for the next day, and then in the evening, add how I moved that day and then see whether it happened, and then add for the next day...then I would know if the goals were low but I moved much more, the steps just appear, just like that, then for the next day I could put a bit higher. (Change talk—taking steps toward change)

P11

Participant 12 realized that adding the activity in the Precious app makes it more likely that she will do the activity the next day:

P12:

Selects an activity

P12: If I already do it [the logging] now, then I have to go there tomorrow in the morning. That’s actually quite good. (Change talk—commitment)

Self-regulation features also received some criticism. Participant 9 expressed sustain change talk on self-regulation, as he thought he is not organized enough to do planning:

I: The meaning is that it could be used for planning, do you think it would work like that?

P9: Ooh, not me at least, I’m so bad at planning anything in advance that I wouldn’t...(Sustain talk—ability not to change) Maybe it could? Maybe it could be used as such. Certainly, if I were a bit more organized person. (Ambivalence)

## Discussion

### Principal Findings

This study presented the development and feasibility testing of the Precious app, which aims to engage users in the behavior change process with relational techniques from motivational interviewing and gamified self-regulation. The feasibility study found that interaction with digitalized motivational interviewing features helped participants reflect their personally valuable goals, needs, desires, and reasons for physical activity. Autonomy supporting features were typically found to be important, although some participants criticized the number of available options and some expressed concerns about being profiled and possibly receiving the wrong types of suggestions. In this section, we will discuss the theoretical framework behind the Precious app and the results of the feasibility study on user engagement in behavior change.

### Theory- and Evidence-Based Development of the Precious App

We developed the Precious app using a theoretical framework (Figure 1), which hypothesized that digitalized elements from motivational interviewing [32] and gamified elements [54] would help to satisfy the basic psychological needs of autonomy, competence, and relatedness, as suggested by the self-determination theory [41,52]. This novel method of combining reflective and spontaneous approaches with motivation and self-regulation has several strengths. This app addresses users with qualitatively different motivation for physical activity and offers support for both reflection of autonomously motivating goals and intrinsically motivating, spontaneous activities. Tailored suggestions provided to users are based on goals, activities, and motivational stages that users have indicated, offering different content to different individuals.

Digitalized motivational interviewing for physical activity has not been previously implemented for smartphones [37], although the need for motivational elements in health apps is recognized [40]. As acknowledged by participants, the Precious app differs from most available health apps, as it does not prescribe goals or behaviors. The choice of exercise as a behavioral strategy (instead of healthy diet, stress management, or sleep hygiene) is made only after a user has decided to aim for an outcome goal that can be achieved through physical activity (Figure 3). The relational features such as empathy, normalization, and affirmations may build a safe environment for change that would not be experienced with standard tracking features alone.

The Precious app’s digitalized motivational interviewing features were implemented as seven question-based tools using seven relational techniques. These relational and motivational features were hypothesized to increase users’ engagement in the behavior change process by increasing relatedness to the app and facilitating self-reflection and the use of self-regulatory BCTs [9], which could help individuals increase health-enhancing physical activity.

High intrinsic motivation is the best predictor of sustained physical activity [44], but not everyone finds physical activity enjoyable as such. Game-based elements can add intrinsically motivating elements to the activity, such as exploring new areas with the *Conquer the city* feature or keeping daily steps up to create a panorama of high mountains with the *Mountain climber* tool.

Control theory–related self-regulatory BCTs were implemented as a *Mountain climber* tool that uses gamification principles to increase intrinsic motivation for BCT use. The self-regulatory BCTs included daily physical activity goal setting, reviewing behavioral goals, making action plans, self-monitoring, receiving feedback on behavior, perceiving the discrepancy between the current behavior and goal, and prompts to complete the daily step goal. These self-regulatory BCTs have been found to increase both physical activity [10] and motivation for physical activity [21], and the gamified visualizations may also appeal to users who would not find self-regulation techniques interesting as such.

### Feasibility Testing

The feasibility analysis of participants’ engagement with the Precious app addressed the following questions: (1) How did participants discuss autonomy support? and (2) what kind of change talk did the Precious app elicit in the users?

#### Research Question 1: How did participants discuss autonomy support?

In line with the self-determination theory, participants valued the autonomy supportive features, such as the tailoring of goals, behaviors, and activities, instead of an externally set behavioral target, as seen in previous Web-based interventions [92,96]. The amount of choice was generally perceived positively, although a couple of participants questioned the high number of options. Some also expressed concerns that their selections might limit their future options. To answer these concerns, we added tutorials emphasizing that all the app functionalities will stay available despite the personalized suggestions and that the suggestions are based on their selections only, which can be changed at any time.

As the Precious app was designed around a list of preselected answer options, it could not offer a complete freedom of choice to the users. A digital service needs to find a balance of the freedom given to the user and the amount of content that can be tailored to users’ wishes. The more determined the answer options are, the more tailored suggestions can be offered to those specific choices. The more freedom the user has in indicating their preferences, the higher is the risk that the app content will not meet those wishes. It is also not known how accurately users want their ideas to be mirrored. Simply rephrasing users’ preferences may not be optimally engaging: One study found an app more engaging when it included recommendations for people resembling the user, instead of recommendations based on personal history only [97]. One option would be an open platform that the users could use to create content for their own needs, creating their own goals, self-regulation techniques, and feedback systems. This type of tool would focus on offering information on possibilities and rationales of using specific techniques, as is done in the *What’s next?* tool of the Precious app.

#### Research Question 2: What kind of change talk did the Precious app elicit in the users?

Participants engaged actively with the app during the think-aloud interviews, making selections and reflecting their choices in relation to their values and current life situation. They expressed a wide range of change talk: mainly *desire*, *reason*, and *need*, when using the motivational interviewing features, and *commitment* and *taking steps toward change*, when using the Mountain climber tool. Considering participants’ varying levels of motivation and activity, it is promising that change talk was identified across the full sample. There were very few occasions of sustain talk and ambivalence. This may be a positive indicator about the ability of the Precious app to evoke positive cognitions about physical activity. The Precious app, however, has limited means for exploring possible negative elements related to increased physical activity or positive aspects of maintaining inactivity, which are often identified in face-to-face counseling [32]. One option for addressing sustain talk would be offering a problem-solving tool with strategies to overcome hurdles, as done by Friederichs et al [73,74].

Surprisingly, the *Time machine* tool for using mental rehearsal of past and future behavior did not elicit change talk during use. As the tool was tested by only two users, it is too early to conclude that this tool would not support other participants. On the basis of a fast pace with which participants advanced through all the Precious app features, more structured guidance (selecting options, typing answers, or listening to instructions) may work better on a smartphone platform than a list of open questions and imagination tasks.

Analyses of the think-aloud walk-throughs underlined how making selections while using the app is part of the discourse on change or a form of *implicit change talk*. The CLEAR coding system, used for recognizing change talk [94], suggests that when participants select numbers with tools such as the Importance and *Confidence rulers*, change talk should only be coded if the participant provides a verbal qualifier for the number. At the first stages of analysis, we aimed to extend this principle to all the interactions with the Precious app, analyzing only clear oral statements. Soon, we discovered that participants’ selections were so embedded in the discourse that including interactions with the service was indispensable to get an accurate picture of the participants’ thought processes.

The *implicit change talk* (ie, making selections that indicate exercise intentions) may differ from change talk that is elicited verbally in interactions with a coach or counselor. Miller and Rollnick [32] point out the difference of reactions when just thinking about questions silently, writing down the answers, or saying them to someone else. Although interacting with an app might not fully correspond to a face-to-face counseling, our results indicate that users can be encouraged to actively reflect options with a digital service. However, it should be noted that the think-aloud walkthrough procedures were undertaken in the presence of a researcher, which might have affected participants’ reactions.

Offering a service with relational elements and tailored feedback may exceed the impact of simply writing down an answer, but it is possible that the relatedness experienced with the service moderates this effect so that the more strongly the user feels an app is an active, responsive body in the interaction, the stronger the impact of the intervention is. The more autonomous and interactive digital services become, the more users may feel responsible for and committed to the actions negotiated with them. Getting users to select answers on their personal choices, values, and achievements may also increase their engagement with the app via activating users’ representations of their identity and self-image. In gaming research, players have experienced digital games to be most intrinsically motivating when their gaming experiences have been congruent with their ideal selves [98].

### Limitations

The think-aloud interviews only assessed a brief (30-60 min) use of the Precious app features. Participants did not have the chance to see how their physical activity accumulates over time or how the app’s feedback messages depend on their goal progress. Thus, the interview only provided information on participants’ initial reactions to the app’s motivational and self-regulatory features. For this reason, despite studying perceived autonomy support, the interviews did not address the two other basic needs of the self-determination theory (ie, competence and relatedness), as we hypothesized these to increase through regular use of the app, with the rules engine providing encouragement and feedback on behavior. Similarly, the gamified elements can only be studied after extended use in the natural environment.

The features of the Precious app were still being developed through the feasibility interview phase, meaning that some features were available for the last interviewees only. *What’s next?*, *Time machine*, and *Confidence ruler* were tested by only two participants because of late implementation. As is typical for pilot studies [27], the sample size is a potential limitation for generalizing the results to a wider population. Future studies could use larger samples of individuals of a certain age, activity level, or technology literacy to provide a broader representation of different types of users.

### Future Directions

Interaction with technology needs further focus on the behavioral sciences. Despite the relational nature of motivational interviewing, studies offering digital motivational interviewing features rarely discuss how accurately it can be delivered with technology and how complexities related to digitalization of motivational interviewing might be addressed [37]. Current definitions of BCTs primarily relate to interactions between humans [8]. In future work, we need to define if digital services can be considered active participants in interactions. For instance, considering the technique *Review behavior goal(s) jointly with the person* [8], is the reviewing done *jointly* if it is suggested by a digital tool or a chatbot and is a digital service with relational elements able to provide *Social rewards*? [8]. These questions need addressing when artificial intelligence becomes increasingly widespread: at what point does an interactive, chatbot-type service start to be a companion in decision making?

The current implementation of the app is mostly text based. Several elements of gamification could be taken further, for instance, the Time machine tool offers a rich platform for visual elements that would enhance the user experience [80]. However, adding complex graphics does not automatically increase user engagement, and text-based elements have been found as effective in increasing physical activity as a digital avatar [99]. In addition, the level of service customization is currently tied to the user’s time and patience to fill in self-evaluations. Evolving technologies offer increasing opportunities to automatically detect and tailor to user emotions [100], depression [101], stress [102], and even sarcasm [103]. Within the Precious app project, the use of social media posts for mood detection was examined but was not included in the current version [104]. These techniques offer promise, but more evidence is needed, whether gamified or chatbot-type interactive content engages users more in the behavior change process than text-based solutions.

### Conclusions

This paper described the development and feasibility testing of the Precious app. We suggested that an app can support engagement in the behavior change process through two pathways: encouraging active reflection of users’ motivations, capabilities, and opportunities for physical activity and providing gamified elements with challenges and fun. Both pathways can satisfy users’ psychological needs of autonomy, competence, and relatedness, increase their autonomous motivation, and support their self-regulation.

The novelty of the Precious app is in providing relational support and addressing both reflective and spontaneous ways of motivating users. The Precious app aims to support an active cognitive engagement with the behavior change process using reflection tasks. The app builds relatedness with relational techniques from motivational interviewing and by supporting basic psychological needs described in the self-determination theory. Gamification elements include the self-regulation tool, which helps users to set goals, make plans, and monitor their progress with a mountain visualization. To collect and present physical activity data accurately, the app aggregates data from several sources: activity bracelets, smartphone sensors, and self-reports. The app consists of independent tools that can be switched on and off, enabling tailoring of personalized suggestions and testing specific intervention elements in a controlled way.

The feasibility interviews of the digitalized motivational interviewing features revealed that participants value personalization options, but the app also needs to clearly communicate how the information collected from users will be used for recommendations and tailoring and which choices users are able to change later. During the think-aloud interviews, motivational app features elicited change talk on needs, reasons, and desires to change, whereas self-regulatory features elicited commitment and taking steps toward change types of change talk. This active engagement in self-reflection can be perceived as a proxy for engagement in the first steps of the behavior change process. The fast pace with which users typically advanced through the app suggests that smartphone-based interventions may benefit from interactive functions instead of open questions and imagination tasks.

Apps supporting behavior change need to engage users in the behavior change process. Feasibility tests with the Precious app suggest that this can be done by addressing users’ psychological needs and by supporting active self-reflection. The impact of specific intervention elements on individuals’ self-regulation and daily physical activity over time will be tested in a series of N-of-1 field studies [105].

## Appendix

Multimedia Appendix 1 ‘How to get there?’ tool for linking users’ favorite activities and outcome goal.

Multimedia Appendix 2 Journey view, map of gamified achievements.

Multimedia Appendix 3 Table on participants’ characteristics.

## Abbreviations

API: application programming interface
BCT: behavior change technique
CLEAR: Client Language EAsy Rating
MET: metabolic equivalent
PRECIOUS: PREventive Care Infrastructure based On Ubiquitous Sensing
RQ: research question
