# Supplementary figures and images for: Engaging Users in the Behavior Change Process With Digitalized Motivational Interviewing and Gamification: Development and Feasibility Testing of the Precious App
Source: JMIR Mhealth Uhealth. 2020 Jan 30;8(1):e12884. doi: 10.2196/12884 (PMC7055776; doi:10.2196/12884)

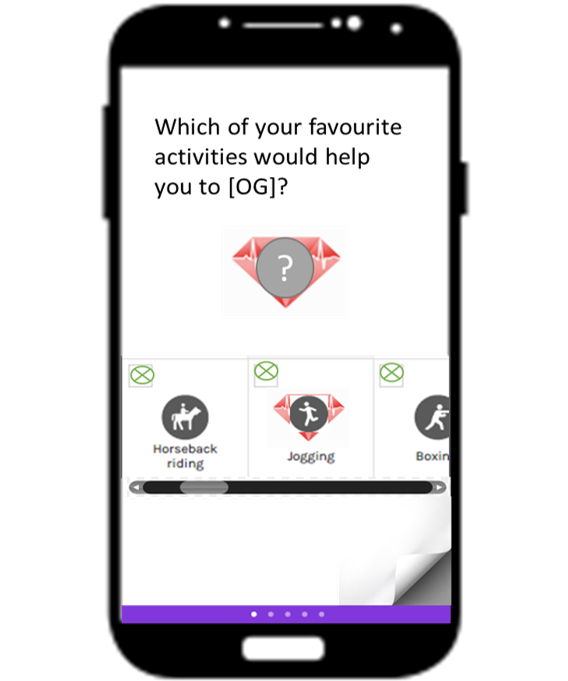

Supplement: Multimedia Appendix 1 [file mhealth_v8i1e12884_app1.png]

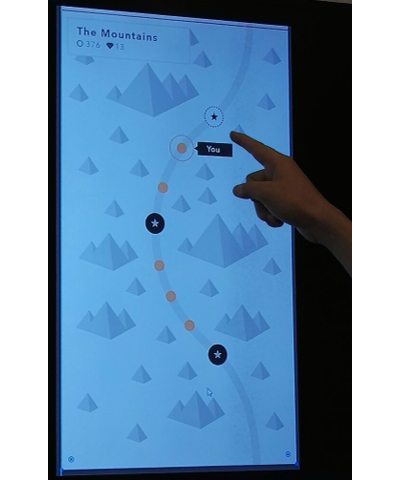

Supplement: Multimedia Appendix 2 [file mhealth_v8i1e12884_app2.png]
