# Supplementary material for: Engaging Users in the Behavior Change Process With Digitalized Motivational Interviewing and Gamification: Development and Feasibility Testing of the Precious App
Source: JMIR Mhealth Uhealth. 2020 Jan 30;8(1):e12884. doi: 10.2196/12884 (PMC7055776; doi:10.2196/12884)
Supplement: Multimedia Appendix 3 [file mhealth_v8i1e12884_app3.docx]

| **Participant** | **Gender** | **Age** | **Physical activity below or above recommendations** [89] | **Thinks that moves enough** | **Familiarity with smartphone apps, 1 = low, 3 = high** | **Educational background** |
| --- | --- | --- | --- | --- | --- | --- |
| **P1** | F | 60-69 | Below | No | 1 | Professional degree |
| **P2** | M | 30-39 | Above | No | 3 | Bachelor’s degree |
| **P3** | M | 30-39 | Above | No | 3 | PhD |
| **P4** | F | 20-29 | Above | No | 3 | Bachelor’s degree |
| **P5** | M | 20-29 | Above | - | 3 | Master’s degree |
| **P8** | M | 30-39 | Below | Yes | 1 | Vocational degree |
| **P9** | F | 20-29 | Above | Yes | 1 | Master’s degree |
| **P10** | M | 20-29 | Above | Yes | No smartphone | High school degree |
| **P11** | F | 20-29 | Below | No | 1 | Master’s degree |
| **P12** | F | 20-29 | Above | No | 2 | High school degree |

Note. Supplementary table 1. Participant details. P6 and P7 completed think-aloud of the biofeedback report only and thus were not included in the analyses.

F: Female; M: Male
